# Maternal Experience Leads to Lasting Gene Expression Changes in Some Regions of the Mouse Brain

**DOI:** 10.1534/g3.119.400249

**Published:** 2019-06-04

**Authors:** Michelle N. Arbeitman

**Affiliations:** Department of Biomedical Sciences, College of Medicine, Florida State University, Tallahassee, FL 32306

**Keywords:** Behavior, transcriptome, mouse, pregnancy, maternal, brain

## Abstract

Rodent maternal behaviors are due to the coordinated effects of fluctuating hormones, with their onset triggered by interactions with newborn pups. Previous studies have shown that many genes have changes in expression during peripartum stages. However, it is unclear if there are long-lasting changes in gene expression, well after the performance of maternal behaviors, that could influence physiology and behavior throughout the remaining lifespan. Here, gene expression differences were examined in mouse between age-matched virgin and primiparous females, at least 4 weeks after weaning. Of the five brain regions examined—hypothalamus, hippocampus, cortex, cerebellum, and the amygdala—only the hypothalamus had thousands of genes with significant expression differences. The cerebellum had 130 genes with expression differences, and the other brain regions had no significant changes detected. The expression changes in the hypothalamus include an enrichment of genes that could mediate long-lasting behavioral and physiological changes, given their known roles in parental behavior, including *galanin* and *prolactin receptor*.

Studies in mammals have shown that there are long-lasting changes in the brain due to maternal experience that are apparent throughout the lifespan (reviewed in Kinsley and Amory-Meyer 2011; Kinsley *et al.* 2012; Hillerer *et al.* 2014; Bridges 2016; Stolzenberg and Champagne 2016). These changes include altered neuronal connectivity, neuron/glial cell number differences, changes in neuronal physiology, hormone levels, and changes that lead to altered gene expression, such as chromatin modifications. Furthermore, in humans, a prospective study of primiparous and nulliparous women found changes in gray matter structure that persisted for at least two years after pregnancy, in regions of the cerebral cortex that are thought to be involved in social processes (Hoekzema *et al.* 2017). Thus, cell- and molecular-level changes due to reproductive experiences may underlie long-lasting behavioral changes that have been observed in mammalian models.

For example, in rats, experienced mothers show a more rapid onset of maternal behaviors to pups from subsequent litters and increased maternal aggression to male intruders (Moltz and Robbins 1965; Scanlan *et al.* 2006). Maternal experience in rats also leads to long-lasting improvement in spatial learning and foraging (Pawluski *et al.* 2006a; Pawluski *et al.* 2006b), and to changes in anxiety levels associated with motherhood (reviewed in Byrnes and Bridges 2006; Lonstein 2007; Macbeth and Luine 2010). In mouse, the experience of pregnancy also influences subsequent behavior, given changes observed in a pup retrieval assay and in an operant conditioning assay in post-partum females compared to virgins (Gandelman *et al.* 1970; Hauser and Gandelman 1985). The study presented here seeks to determine if there are changes in gene expression in the brain that could underlie these long-lasting behavioral changes in mouse.

Previous studies have shown that thousands of mouse genes have changes in expression during peripartum stages (Gammie *et al.* 2005; Gammie *et al.* 2016; Ray *et al.* 2016). Our previous study showed that the changes were both time point and brain region specific (Ray *et al.* 2016). The KEGG pathway enrichment analyses results suggested that pregnancy and parturition results in a major developmental transition in the brain (Ray *et al.* 2016), given many pathways with known developmental roles had changes in gene expression. A remaining question is whether any of these gene expression changes are long-lasting and/or generate long-lasting changes in expression of other genes, well after the performance of maternal behaviors. Identification of these genes could provide mechanistic insight into the known long-lasting behavioral changes that are due to reproductive experience.

Gene expression differences were examined between age-matched virgin and primiparous female mice (their first litter)—greater than four weeks after weaning. In the five brain regions examined—hypothalamus, hippocampus, cortex, cerebellum, and the amygdala—only the hypothalamus had thousands of genes with significant expression differences. The cerebellum had 130 genes with expression changes, whereas the other regions had no detectable, significant changes. Gene expression in the hippocampus, cortex, and amygdala in primiparious females is similar to that of nulliparous females. While robust differences in gene expression were evident throughout the brain during peripartum periods (Ray *et al.* 2016), the gene expression landscape four weeks after weaning in primiparous females is more similar to virgins.

## Methods

### Animal husbandry

The animal work was done by Jackson Laboratory (Bar Harbor, ME). The genotype of all animals is C57BL/6J (stock # 000664, Jackson Laboratory). Tissue was harvested from age-matched virgin and primiparous females (breeder females), all born on the same day +/− 3 days. Supplemental Table 1 includes information on mouse ID#, age, breeding, litter date of birth, size and sex of pups, date weaned and date to necropsy.

All animals in this study were housed in the same animal barrier room, on the same light/dark cycle. The unmated females were housed eight per cage. For mated females, one male and one 7.5–8-week-old female were housed per pen for two weeks and then the males were removed. In this experiment only the primiparous/breeder females experienced social interactions with males and were mated with males, so some of the changes in gene expression detected could be due to these additional experiences.

The date the litters were born for the females in this study was the same day +/− 3 days (Supplemental Table 1). The litter size ranged from 4–8 pups, with a mean of 6.2 +/− 1.4 pups (upper 95% mean =7.3, lower 95% mean = 5.2) (Supplemental Table 1). The pups were weaned 22–26 days after their date of birth. Tissue was harvested from primiparous females 33–41 days post-weaning (Supplemental Table 1). The range of days ensured all tissue was harvested on the same day and that all females were at least 30 days post-weaning. The rationale for choosing these time points was to not wait too long, to ensure that the study detects changes due to reproductive experience rather than aging, given the short life span of the mouse. The other consideration was that the interval was sufficiently long-enough after weaning to determine if there are gene expression changes that are independent of having actively performed maternal behaviors. This study serves as a starting place to examine gene expression post-reproductive experience and additional time points can be added based on the results here.

Tissue from virgin and primiparous females was harvested on the same day, snap frozen in RNA later solution (Ambion) and harvested within a two-hour window. Frozen brain tissue was shipped to Florida State University on dry ice. The brain regions analyzed in this study are the hippocampus, hypothalamus, amygdala, cerebellum and the cortex. The entire hippocampus, hypothalamus, amygdala, cerebellum and cortex was dissected, but with the exception of the hypothalamus (entire region was used) only the left side of the region was used in this experiment. All tissue was kept frozen at -80° until RNA purification. Euthanasia was by cervical dislocation, without anesthesia. All animal work was done by Jackson Laboratory (Bar Harbor, ME) in accordance with the guidelines of the American Association for the Accreditation of Laboratory Animal Care (AAALAC). The protocol conducted in this study was approved by the Jackson Laboratory and Florida State University Animal Care and Use Committees.

**Figure 1:**
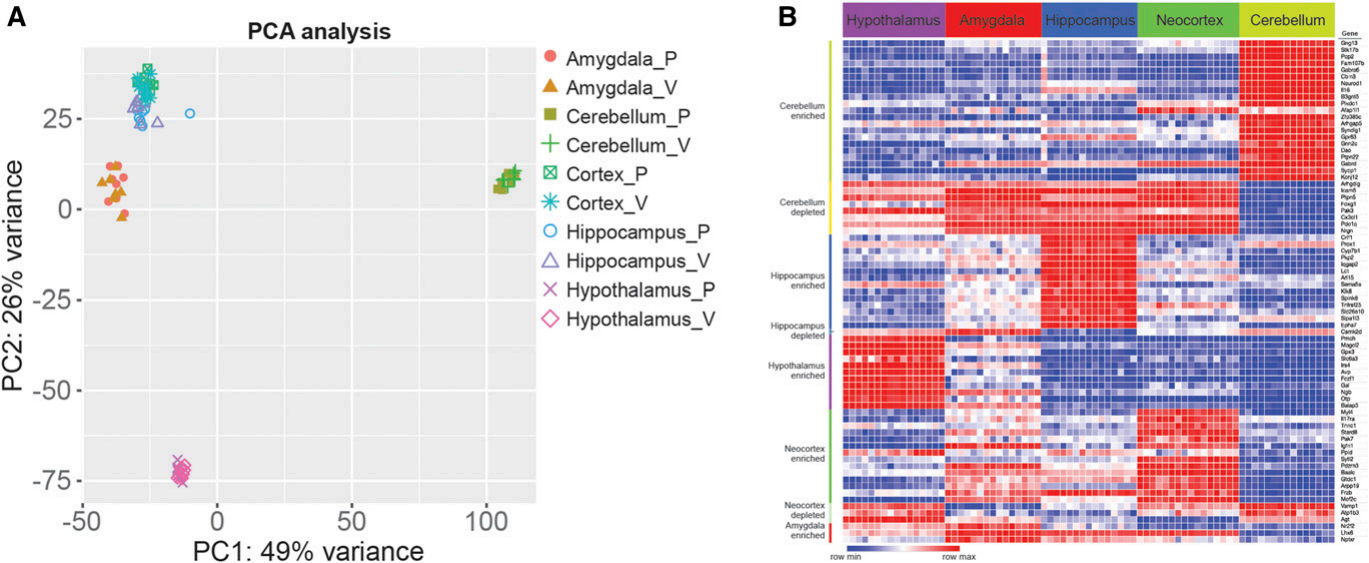
Gene expression in the female mouse brain in virgin and primiparous females. (A) Principle component analysis of the data. The data are color coded to indicate the brain region and reproductive state (P for primiparous, V for virgin). (B) Heatmap of a set of genes with known enriched or depleted expression in each brain region (made with Morpheus: https://software.broadinstitute.org/morpheus).

In Figure 1, validation of the brain region dissections and library preparation was performed by examining expression of genes with known region-specific patterns, as determined by previous studies (Sandberg *et al.* 2000), and through identifying brain-region-specific genes from the Allen Brain Atlas (Lein *et al.* 2007). The gene list is provided (Supplemental Table 2).

### RNA-seq library preparation, sequence read mapping and analysis

RNA and protein were extracted from the frozen brain tissue using the NucleoSpin RNA/Protein purification kit (Macherey-Nagel). The brain tissue was homogenized using a motorized homogenizer and 1 ug of total RNA was used for subsequent mRNA isolation (New England Biolabs Inc. PolyA magnetic mRNA isolation kit). The External RNA Controls Consortium (ERCC) RNA Spike-in Controls (Ambion 4456740) were diluted 1:100 and added to total RNA before mRNA isolation. RNA-seq libraries were generated using the NEBNext Ultra RNA Library Prep Kit for Illumina (New England Biolabs Inc. E7530L). The quality of the libraries were examined using Agilent High Sensitivity DNA Bioanalyzer Chips (Agilent Technologies 5067-4626) and quantified by KAPA Library Quantification Kits for Illumina sequencing platforms (KAPA Biosystems KK4824). The libraries from each brain region were pooled together for sequencing on the Illumina 2500 platform to obtain single-end, 100 base reads.

Eight biological replicates were used in this study for each condition, with each library replicate made from mRNA from a single mouse, for a total of 80 libraries. The average number of reads/library for each brain regions is: hippocampus (30 million reads/library), cerebellum (25 million reads/library), cortex (27 million reads/library), hypothalamus (20 million reads/library), and amygdala (19 million reads/library). Quality checks on the sequencing data were performed by FastQC and all libraries passed this initial check. Read mapping was performed using Hisat2 and the UCSC reference genome mm10 from the Hisat2 webpage (Kim *et al.* 2015), on the Florida State University High Performance Computing cluster. Alignment rates for all libraries was 90% or greater. The featureCounts function within Rsubread package was used to obtain count tables (Liao *et al.* 2019), using the inbuilt mm10 annotation files, to obtain meta-feature level ‘reads that map a single time’.

Count tables were imported into iDEP to inspect the data, pre-process and perform initial analysis (Ge *et al.* 2018). An initial meta-analysis of data from all brain regions (80 libraries) showed clear partitioning of the data based on brain region, with three outlier samples removed based on principle component analysis (1 sample from the hippocampus, cerebellum and amygdala data sets). The data were then pre-processed, with a minimum of 5 counts per million (CPM) in at least 8 libraries, for a gene to be further analyzed. This cut-off was chosen based on visualization of the expression values, where a bimodal distribution was observed. The count data were transformed, using EdgeR: log2(CPM+c), with pseudocount c =4. For differential expression analysis, limma-voom was used within the iDEP workspace. The principle component analysis, heatmaps and k-means cluster analyses were also performed in the iDEP workspace and the R code used for these analyses are provided (Supplemental Table 2). Gene set enrichment analysis was performed in ShinyGO (http://bioinformatics.sdstate.edu/go/) (Ge and Jung 2018).

In the Figures and Tables the following abbreviations are used for brain region: hypothalamus (Hypo), amygdala (Amy), hippocampus (Hippo), cortex (Ctx), cerebellum (Cer). Mating status is abbreviated: virgin (V) and primiparous (P). The mouse ID number is linked to all data samples. The processed and raw count data, validation genes, and statistical analyses are provided (Supplemental Table 2).

### Data Availability

All raw data are available through NIH GEO via accession number GSE125428.

Supplemental Table 1 contains mouse husbandry information. Supplemental Table 2 contains processed and unprocessed count data, statistical analysis, validation genes, and Rcode. Supplemental Table 3 contains lists of genes from k-means cluster analysis shown in Supplemental Figure 1; KEGGS and GO analysis of genes in each cluster. Supplemental Table 4 contains KEGG pathway and GO analysis with lists of genes with significant differences in hypothalamus and cerebellum. Supplemental material available at FigShare: https://doi.org/10.25387/g3.8014703.

## Results and Discussion

### Meta-analysis of data from all five brain regions

Gene expression analysis was performed using RNA-seq, from tissues derived from eight virgin and eight primiparous females that were > four weeks after pup weaning. Expression was examined in the hypothalamus, hippocampus, cortex, cerebellum, and the amygdala. A principle component analysis (PCA) of the data shows that samples from the same brain region cluster together, irrespective of reproductive status (Figure 1A). For each brain region, the data from virgin (V) and primiparous (P) females is highly overlapping in the PCA plot. The hippocampus and cortex had the most similar gene expression levels, with the amygdala data next most similar to the data from these two regions (Figure 1A). This is consistent with our previous study that found expression in the hippocampus and neocortex were most similar, irrespective of the peripartum time point examined (Ray *et al.* 2016). In the PCA plot, the data from the hypothalamus and cerebellum were the most distinct from data from the other brain regions (Figure 1A). To validate the dissections and library preparations were performed correctly, an examination of genes with known enriched or depleted expression in the brain regions was performed, using a heat map to visualize (see methods). Genes show the expected expression patterns (Figure 1B; Supplemental Table 2 **for gene list).**

An additional clustering analyses was performed to examine how related the expression patterns in brain regions are based on the 1,000 genes with the most variable expression. The results are similar to those based on PCA analysis (Supplemental Figure 1A)—the data from both the virgin and primiparous brain regions, for each brain region, cluster together. A *k*-means cluster analysis of the top 1,000 variable genes (10 clusters; Supplemental Figure 1B) shows that clusters of genes with high expression in a brain region are enriched with expected Gene Ontology (GO) and KEGG terms, based on known functions of the brain regions (GO and KEGG analysis see: Supplemental Figure 1-5 and Supplemental Table 3) (Ashburner *et al.* 2000; Kanehisa *et al.* 2016; Kanehisa *et al.* 2017). For example, genes in Cluster C have high expression in the cerebellum and are enriched with genes that have GO (Biological Process) term ‘*Cerebellar cortex morphogenesis*’. Furthermore, genes in Cluster D have high expression in the hypothalamus and are enriched with genes that have the GO Biological Process term ‘*Feeding behavior*’.

### Brain-region-specific expression analysis

Next, gene expression differences between age-matched virgin and primiparous females were examined within each brain region. Genes with expression differences in this study will be referred to as genes with long-term expression differences. The hypothalamus was the only brain region that had thousands of genes with long-term expression differences (2,660 genes), whereas the cerebellum had fewer (130 genes). The hippocampus, cortex, and amygdala had no apparent, significant, long-term differences (FDR corrected *p* value cut-off of *q* <0.05). For genes with expression differences, the average fold-change was small (hypothalamus, mean = 1.03+/−0.02; cerebellum, mean = 1.04+/−0.02), with no genes showing > twofold expression differences (Supplemental Table 2).

#### Hypothalamus:

The hypothalamus is a link between the endocrine system and the nervous system, acting as the central nervous system relay point in the hypothalamus-pituitary-gonadal axis. Thus, the hypothalamus has important functions in pregnancy and parturition, as it is responsible for releasing and responding to hormones that drive progression through pregnancy. The hypothalamus’ other functions include thermoregulation, satiety, feeding, circadian rhythms, and blood pressure, among others (reviewed in Brunton and Russell 2008; Brunton and Russell 2010). If we consider the virgin reproductive state baseline in this comparison, genes with higher expression in virgin females are repressed by reproductive experience and genes with higher expression in primiparous females are induced by reproductive experience. In the hypothalamus, 2,660 genes had significant differences in expression, with 1,608 genes repressed (higher in virgin females) and 1,052 genes induced (higher in primiparous females) by reproductive experience.

**Figure 2:**
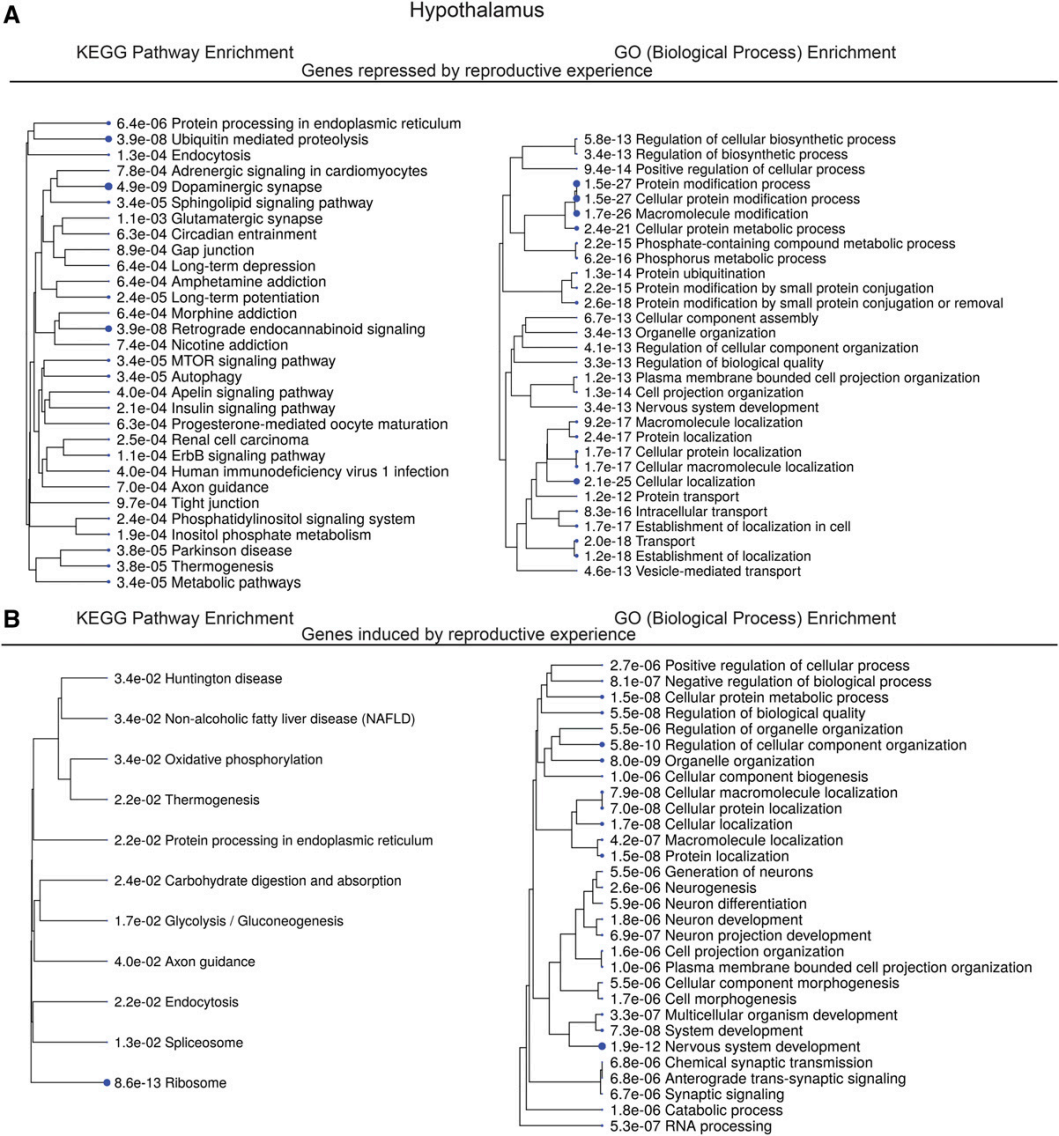
KEGG pathway analysis of genes with differential expression in the hypothalamus. Tree view of KEGG pathway and GO (Biological Process) term enrichments for genes with significantly long-term repressed (A), and long-term induced (B) expression changes.

An examination of the KEGG pathways shows that genes that are repressed or induced by reproductive experience in the hypothalamus include those with neuronal functions, as well as other functions (Figure 2) (Kanehisa *et al.* 2016; Kanehisa *et al.* 2017). There are KEGG pathways that are in common to the long-term repressed and induced genes, including ‘*Processing in the endoplasmic reticulum*’, ‘*Endocytosis*’, and ‘*Axon Guidance*’. A gene can appear in only one of the lists, so different genes in these pathways are induced and repressed long-term, which can be expected in pathways with both positive and negative factors. Both the induced and repressed lists also share GO (Biological Process) terms, including ‘Nervous system development’, and ‘Macromolecular localization’.

From our previous study (Ray *et al.* 2016), if we examine the KEGG pathway enrichments for the genes that change expression in any pair-wise comparison between virgin and the peripartum stages examined (Supplemental Figure 6), we find no overlap with the KEGG pathway enrichments for the genes that induced and repressed long-term, based on the criteria used here. However, the long-term induced and repressed gene lists, and the genes with expression differences in Ray *et al.* are enriched with genes annotated as functioning in GO (Biological processes): ‘Generation of neurons’ and related terms for neurogenesis. Taken together, peripartum and long-term expression changes in the brain do involve gene expression changes that underlie common ‘nervous system function’ and other cellular processes in terms of GO annotation.

Furthermore, in our previous study of virgin and peripartum stages, we found ten genes that maintain repressed expression relative to virgins, but no genes that maintained induced expression (Ray *et al.* 2016). Only one of the ten repressed genes, *D site albumin promoter binding protein* (*Dbp*), had significant, long-term expression differences. Here, *Dbp* is induced long-term by reproductive experience, rather than repressed. The Dbp transcription factor is known to underlie circadian rhythms (Ripperger *et al.* 2000), though it is not known if there are long-term circadian rhythm changes due to reproductive experience.

An examination of the top five most significant KEGG pathways for genes that are repressed long-term reveal: ‘Dopaminergic synapse’, ‘Ubiquitin mediated proteolysis’, ‘Retrograde endocannabinoid signaling’, ‘Protein processing in endoplasmic reticulum’, ‘Long-term potentiation’ (Figure 2, Supplemental Table 4, Supplemental Figures 7-11). The top five pathways for genes with higher expression in primiparous females include: ‘Ribosome’, ‘Spliceosome’, ‘Glycolysis / Gluconeogenesis’, ‘Protein processing in endoplasmic reticulum’, ‘Endocytosis’. (Figure 2, Supplemental Table 4, Supplemental Figures 12-16). Given the pathways identified it appears there are long-lasting changes in several signaling pathways that are specific for neurons, and also in cellular machinery/organelles that are found in most cell types.

In our previous study we compiled a list of 72 genes with known roles in pregnancy and/or reproduction (see Supplemental Table 25 in Ray *et al.* 2016). There are six genes that show long-term repression of expression and overlap with these 72 genes: 1-3) *GABA A receptor*, *subunits alpha 1,2 and 3* (*Gabrg* 1, 2 and 3); 4) *serotonin receptor 2C* (*Htr2c*); 5) *neurotransmitter transporter*, *dopamine* (*Slc6a3*); and 6) *prolactin receptor* (*Prlr*). On the other hand, there are three genes that show long-term induction of expression and overlap with these 72 genes: 1) *prolactin regulatory element binding* (*Preb*); 2) *galanin* (*Gal*); and 3) *neuropeptide Y* (*Npy*). Given that these genes have known roles in neuroendocrine functions (Maguire and Mody 2008; Merchenthaler 2010; Angoa-Pérez *et al.* 2014; Wu *et al.* 2014; Muroi and Ishii 2016), they are interesting candidates to functionally analyze to determine their role in long-term behavioral changes. In fact, *galanin*-expressing neurons in the medial preoptic area of the hypothalamus have been found to be necessary for aspects of parental behavior and pup-directed aggressive behaviors in mouse (Wu *et al.* 2014; Kohl *et al.* 2018).

#### Cerebellum:

The cerebellum is known to be a motor control region of the brain and has been included in these studies largely as a control region, as it is not thought to have a specific function in pregnancy and parturition generally. However, there is evidence that the cerebellum has non-motor functions important for many aspects of behavior (Strick *et al.* 2009). In the previous study focused on peripartum time points, 606 genes changed expression in the cerebellum, which was relatively fewer than the other brain regions examined that had more than 1,000 genes with expression changes. Here, 130 genes had significant differences in expression, with 29 genes repressed by reproductive experience (higher in virgin females), and 101 genes induced by reproductive experience (higher in primiparous females). Given the list of genes higher in virgins contains only 29 genes, gene set enrichment analysis does not find significant overrepresentation. An examination of the KEGG pathway enrichment for the 101 genes higher in primiparous females shows the most significant enrichments are ‘*Oxidative phosphorylation*’ and ‘*Parkinson’s disease*’ (Supplemental Figure 17)—many of the enriched KEGG pathways were included largely due to increased expression of genes with functions in the mitochondria. The cerebellum has expression changes with genes that function in a variety of Biological processes (Supplemental Table 4), including ‘Nervous System Development’, which supports the idea that the cerebellum may have more complex roles, including for maternal behaviors, beyond motor control (Strick *et al.* 2009).

#### Brain regions with no expression differences:

The brain regions for which no genes had significant expression differences—hippocampus, cortex and amygdala—are known to underlie maternal experience, learning and memory, and emotional state, among many functions. Even if a reduced FDR cut-off is employed (q < 0.1), no significant expression differences were detected. Future higher resolution neuroanatomical and gene expression studies are required to fully understand if there are long-lasting changes in these brain regions due to maternal experience.

## Conclusions

The observation that the hypothalamus has a large number of expression changes fits well with recent, high resolution functional studies of parental behaviors (Wu *et al.* 2014; Kohl *et al.* 2018). Future studies in the hypothalamus, especially in sub-nuclei that direct maternal behaviors may reveal additional genes with expression changes, especially using new single cell RNA-seq approaches. Furthermore, single cell approaches may reveal that changes in expression are of larger magnitude than observed with bulk RNA-seq and more pervasive, given the averaging of expression values across all cells with bulk RNA-seq might limit detection. Further studies using other model systems will also be informative, to identify and understand the evolutionarily conserved gene expression changes. The rich body of information regarding long-lasting, post-reproductive changes in the rat brain and behavior suggests that expression studies in this model system would add important new insights that can be put into context with rat behavioral studies (Bridges 2016), and also for the identification of evolutionarily conserved changes.
